# Anaesthesia and climate change: time to wake up? A rapid qualitative appraisal exploring the views of anaesthetic practitioners regarding the transition to TIVA and the reduction of desflurane

**DOI:** 10.1186/s12871-024-02693-5

**Published:** 2024-08-30

**Authors:** Syka Iqbal, Amelia Karia, Damon Kamming, Daniel Herron, Luke O’Shea, Cecilia Vindrola-Padros

**Affiliations:** 1 Department of Targeted Intervention, University College London (UCL), Charles Bell House, 43-45 Foley Street, London, W1W 7TY UK; 2 University College London Hospitals NHS Foundation Trust (UCLH), London, UK; 3 Department of Psychology, University of Bradford, Bradford, UK

**Keywords:** Climate change, Sustainability, Climate change mitigation, Anaesthesia, Desflurane

## Abstract

**Background:**

The National Health Service (NHS) has pledged to reach carbon net-zero by 2040. In alignment with this goal, a London hospital’s anaesthesia department is actively reducing desflurane use and transitioning towards total intravenous anaesthesia (TIVA) as a sustainable alternative, contributing to environmentally responsible practices within the healthcare sector.

**Methods:**

We conducted a rapid qualitative appraisal through online interviews with 17 anaesthetic practitioners to explore their perspectives regarding this climate change mitigation strategy. Data analysis was undertaken through the use of rapid appraisal sheets and a framework analysis method.

**Results:**

Participants highlighted the disadvantages of TIVA, including the increased effort, heightened monitoring requirements, operational challenges, and a lack of clinical confidence associated with its use. Despite these reservations, participants acknowledged TIVA’s potential to reduce postoperative nausea. There were perceptions that senior staff members might resist this change due to habits and scepticism over its impact on climate change. To facilitate greater TIVA adoption, participants recommended enhanced training, the implementation of a dashboard to raise awareness of greenhouse gas (GHG) emissions, and the presence of strong climate change leadership within the department. Participants believed that a shift to TIVA should be followed by specific measures such as addressing waste management which is crucial for GHG reduction, emphasising the perceived link between waste and emissions.

**Conclusions:**

The evaluation examines stakeholder attitudes, perceptions, and behaviours, focusing on transitioning from desflurane to TIVA. The study highlights the importance of staff engagement, organisational support, and underscores the crucial role that healthcare practitioners and leadership play in fostering sustainability within the healthcare sector.

**Supplementary Information:**

The online version contains supplementary material available at 10.1186/s12871-024-02693-5.

## Background

According to the World Health Organisation, climate change represents one of the greatest threats to public health [1]. In the UK, the National Health Service (NHS) contributes approximately 4–5% of the total carbon footprint [2], thus inadvertently contributing to the overall impact of greenhouse gases (GHGs) emitted by the health sector [3]. This carbon footprint comprises various GHGs, including carbon dioxide (CO2) and non-CO2 GHGs. While carbon footprint assessments use CO2 emissions as a common measurement and comparator, their global contribution is relatively minor. However, they are important for perioperative emissions reduction. The NHS has pledged to become carbon net-zero by 2040, for the emissions they can control directly. On the 1st of July 2022, this was embedded into legislation [4]. The Delivering a Net Zero NHS strategy is committed to reducing the NHS carbon footprint by 40%, specifically targeting the 2% associated with anaesthetic gases through transforming anaesthetic practice [5]. An important component of this lies in clinicians’ behaviour to avoid or replace carbon intensive practices [6].

The estimated contribution of volatile anaesthetic gases in climate temperatures range from 0.01 to 0.1 of total GHG emissions. However, despite their relatively minor global contribution, these estimations can fluctuate due to inconsistent application of methods and reporting [7]. Desflurane is a scope 1 volatile anaesthetic agent that can contribute to GHG emissions directly and is commonly administered to patients by inhalation during anaesthesia procedures. It is understood that desflurane is the most damaging of these gases and has continued to grow steadily from 2000 to 2018 [8]. However, a recent study has shown that its atmospheric contributions are stable and possibly even started to decline [9]. To quantify the environmental impacts, a study found that desflurane has a 20-fold difference in GHG emissions compared to sevoflurane and 5-fold difference to isoflurane [10]. To further contextualise the damage caused, a single hour of desflurane use at a fresh flow gas rate of 1 L/min is equivalent to the emissions produced by a 370 km drive.

The adoption of Total Intravenous Anaesthesia (TIVA) results in lower environmental impacts of GHG emissions associated with volatile agents, achieved through the use of energy-intensive equipment [11]. Transitioning from desflurane to alternative volatile agents or TIVA can be considered a mitigation strategy [12, 13] with equivalent quality of care [2]. Other volatile agents rather than TIVA can offer significant environmental advantages without some of the potential downsides associated with TIVA, such as increased turnover time, greater waste generation, and added technical complexity [14]. However, it is important to note that TIVA may outperform all volatile agents, in terms of specific outcomes such as reduced postoperative nausea and vomiting and does not lead to patient harm [2].

The perspectives of healthcare workers (HCWs) in strategies to transition from desflurane to TIVA are important as they help understand the attitudes regarding climate change, their knowledge of the subject, addressing specific concerns or challenges and organisational culture to create lasting changes [15]. Some studies have found that HCWs believe there is insufficient knowledge surrounding climate change in hospital environments [16]. In a three-stage Delphi consensus involving 45 anaesthetists globally, all with an interest in sustainability, it was concluded that anaesthetists do not believe that patient safety would be compromised by sustainable anaesthetic practice, with ‘consensus’ defined as over 75%. Additionally, they agreed that hospitals should be mandated to reduce their GHG emissions [17]. Clinical and administrative staff recognise climate change to be a critical issue and identify energy and waste to be the main contributors to GHG emissions. However, they do not feel responsible for mitigating climate change. This perception stems from their belief that prioritising patient care should be the utmost of their priorities [18]. Consequently, it is widely recognised that interventions tackling clinicians are the most effective and thus studies exploring the disparity between attitudes and behaviour are needed to reduce barriers to climate change mitigation strategies [19].

This study focuses on the transition from desflurane to TIVA as one strategy that anaesthetic practitioners can employ to reduce GHG emissions. This intervention consists of two sub-interventions (1) phasing out desflurane compared to other volatile anaesthetic agents with less greenhouse warming potential, (2) changing from volatile anaesthetic agents to TIVA. It’s important to note that the perception, barriers, and enablers of each sub-intervention might be vastly different.

### Aims

In this evaluation we explore the views of anaesthetic practitioners at a London hospital, to identify the advantages and disadvantages of this transition, as well as potential barriers, enablers and recommendations.

This study was guided by the following research questions below:

- How do anaesthetic practitioners perceive the reduction of desflurane in relation to their practice?
- What are the perceived barriers and enablers to phasing out desflurane and increasing TIVA anaesthesia?
- What strategies can be employed to effectively educate and engage anaesthesia practitioners in sustainable anaesthesia practices?

## Methods

### Design

This study was designed as a rapid qualitative appraisal, with interviews as the main source of data [20]. A rapid qualitative appraisal design is recommended to collect and analyse data in a targeted manner, under limited timeframes, to ‘diagnose’ situations [21]. Given the rapidly evolving healthcare interventions, real-time data collection and analysis are useful to support quicker implementation [22, 23]. These methods tend to rely on the use of teams to collect and analyse data in short timeframes [24].

### Sample and recruitment

Purposive sampling was used to ensure diversity of anaesthetic practitioners from a London hospital. The sample included 17 participants, including consultant anesthetists (*n* = 11), registrars (*n* = 3), trainees (*n* = 2) and anesthesia associates (*n* = 1). Staff members were sent an initial email with a participant information sheet (PIS) and given time to review and ask questions. Throughout recruitment, the evaluation team carefully reviewed the sample. A rigorous sampling framework targeted different levels of seniority and ethnic identities. Recruitment was limited by participant availability and willingness to participate. Interviews were arranged via email communication and all of the recruited participants provided written consent.

### Participants characteristics

Participants employed in the London anesthetic department took part in the study. To protect participants identities, we have not included detailed table of characteristics. The participant group included a range of professional roles, such as consultant anaesthetists, trainee registrars, and anaesthetists. The participants varied in their years of experience, ranging from early career professionals to those with over 20 years of practice.

### Study setting

This study was conducted at a large academic health science centre, with 12,000 employees, and is one of the five comprehensive biomedical research centres in the UK. The hospital organisation has pledged sustainability objectives to achieve Net Zero emissions by 2031. In line with this, the Department of Anaesthesia & Peri-Operative Medicine formed an Anaesthesia Climate Emergency (ACE) Team to drive the department to Net Zero Anaesthesia. The department is a large academic department which comprises more than 150 consultant anaesthetists.

### Data collection

In-depth, semi-structured interviews were conducted with staff members from the anaesthetics department at a London hospital via Microsoft Teams from 9 March 2022 to 6 April 2022. This evaluation was undertaken by an external team conducting this study independently from the anaesthesia department or any other clinical unit at the hospital. Interviews lasted between 20 and 30 min, and were conducted by a single researcher, using an interview topic guide (appendix 1). The interviews aimed to capture staff members’ perceptions of climate change, the removal of desflurane, transitioning towards intravenous anaesthesia, reasons why some staff may not support this change, organisational barriers, enablers, and recommendations for climate change mitigation and staff education (see interview topic guide). Prompts were included for questions asked to ensure topics were fully explored [25]. During the data collection process, broader topics of all volatiles emerged. These themes were not indicative of bias but rather a reflection of the participants’ perspectives and the complex nature of the topic. All interviews were audio recorded and the interviewer took notes during the interviews. The study was classified as a service evaluation according to the Health Research Authority (HRA) decision tool, so it did not need to be reviewed by a Research Ethics Committee. Human Ethics and Consent to Participate declarations: All of the recruited participants provided written consent for their participation in the interviews.

### Data analysis

We employed a rapid qualitative data approach for the analysis [21]. We used RREAL rapid assessment procedures (RAP) sheets to structure and summarise data as it was being collected. This method allowed the evaluation team to maintain consistency throughout data collection and help identify the key findings [26] ensuring a high level of inter-rater reliability [27]. To manage potential researcher bias, we implemented several strategies to ensure the credibility of our findings. We used Framework Analysis [28], a form of thematic analysis involving a codebook approach. Analysis involved creating and defining a matrix of themes using a coding template which was developed and refined through an iterative approach. The evaluation team met weekly to discuss and agree on codes, hold discussions on the indexing of codes, and reaching consensus on themes, which facilitated critical reflections and minimised individual biases. Inter-rater reliability was determined by having multiple researchers independently code the same set of data, with discrepancy discussed until a consensus was reached. Additionally, we employed triangulation by cross-verifying data from RAP sheets and recordings to identify any gaps or inconsistencies, enhancing the reliability of our results. Recruitment ended once the evaluation team stopped receiving responses from potential participants. Further recruitment rounds were not conducted as data was considered to have reached saturation by 17 interviews as no new themes were identified. Finally, we followed Rapid and Rigorous Qualitative Data Analysis (RaDAR) techniques to reduce the volume of the qualitative data [29]. This involved creating more concise Microsoft Word tables, a process known as ‘data reduction,’ which transformed textual data into a more manageable and user-friendly format. Quotes were identified by using participants’ professional role to preserve the context of their perspectives.

## Results

The key findings of this study can be categorised into the following themes: Perceptions of ending the use of desflurane and increasing the use of intravenous anaesthesia; the advantages and disadvantages of ending the use of desflurane and increasing the use of total intravenous anaesthesia; the barriers, and enablers of this change and recommendations identified by participants. We describe each theme and present tables with illustrative quotes in the following sections.

### Theme 1: Perceptions of ending the use of desflurane and increasing the use of intravenous anaesthesia

Awareness of this transition within the department varied among staff members. Some participants stated that they did perceive it as a significant shift in practice since desflurane is not used in theatres anymore, whilst others claimed they were unaware that this change was underway.

*“It is not discussed in the department and not considered a critical issue”* [Consultant Anaesthetist].

Exploring how staff felt about the transition revealed conflicting responses. Some believed it was a positive change, as the negative impacts of volatiles on the environment were well-established and they were very willing to shift completely towards TIVA. *“Anaesthetists are the most open to ideas and willing to make changes”* [Anaesthetic Registrar].

However, others expressed concern about getting used to changing their standard technique. A few participants were against going vapour-free and stated there are no right or wrongs in anaesthesia, only different ways of doing things, and by reducing their choice, their usefulness as clinicians is limited.

*“There is just different ways to do things. It’s nice to have a variety of methods and by reducing our choice*, *it limits how useful we can be. So*, *I am a little against going for a zero-vapour situation”* [Consultant Anaesthetist].

### Theme 2: Advantages and disadvantages of ending the use of desflurane and increasing the use of intravenous anaesthesia

Participants discussed advantages of this transition, including improved patient outcomes and organisational benefits such as a faster turnover of patients. However, disadvantages were frequently mentioned, such as patient awareness, the challenges associated with the maintenance of pumps whilst using TIVA, the dangers of clinicians’ lack of confidence with the technique and the increase in single-use plastic as a result of using TIVA. Tables 1 and 2 presents participants’ perceptions of the advantages and disadvantages of this climate change mitigation strategy with supporting quotes.

**Data Table 1:** Participants perceptions of the advantages of this climate change mitigation strategy and supporting quotes

Advantages	Quotes
**Evidence basis and research**	
Staff were aware of the greenhouse gas emission reduction benefit of this change. They conveyed that there is strong evidence basis behind the environmental benefits of TIVA.	*“Now that we have the added knowledge that the inhalation agents have negative impacts on climate change*, *it has added another reason as to why I want to use TIVA”* [Consultant Anaesthetist]. *“I read somewhere that using desflurane is like flying to New York from London with only 6 people on the plane- so*, *I’m not sad to see it go”* [Anaesthetic Trainee].
**Patient care**	
Participants frequently mentioned the advantage of TIVA in providing better patient experiences and post-operative outcomes.	*“Smoother anaesthetic*, *less haemodynamic changes*, *better intraoperative analgesia*, *patients wake up smoother*, *they cough and vomit less and have less of that hungover effect”* [Trainee Registrar]. *“TIVA offers predictability and stability in terms of the wake-up profile […] this made me want to use TIVA for everyone”* [Consultant Anaesthetist].
**Advantages of specific types of surgery**	
Participants reported that TIVA worked better for airway, head and neck, and ENT surgery.	*“TIVA really suits the style of anaesthetic needed for ENT and head and neck surgeries*, *there’s that added safety profile”* [Consultant Anaesthetist].
**Organisational benefits**	
In terms of organisational benefits, the faster turnover of patients due to fewer post-operative complications will lead to a positive impact on bed flow.	*“Organisationally*, *there are advantages*, *you could enable quicker turnaround*, *as less complications means patients are in recovery for less time”* [Anaesthetic Registrar].

**Data Table 2:** Participants perceptions of the disadvantages of this climate change mitigation strategy and supporting quotes

Disadvantages	Quotes
**Patient awareness**	
Increased chance of patient awareness and lack of reliability of brain monitoring.	“*There is a constant worry about awareness […] there is no machine to tell us we have achieved anaesthesia with the level that has been administered”* [Consultant Anaesthetist].
**More intensive monitoring**	
TIVA requires maintenance of pumps, continuous drawing up of drugs, constant vigilance of connections and infusions, and tailoring of dosage to patient demographics.	*“TIVA is a lot more technical*, *a lot more work and lot more anxiety” [Consultant Anaesthetist].* *“It leaves more space for error because you have more things to consider” [Anaesthetist].*
**Operational insufficiencies**	
The limited accessibility of infusion pumps and brain monitoring equipment was challenging.	*“A major issue is a lack of availability of infusion pumps*, *my first job before I even saw patients would be to find the pumps*, *hide them in a cupboard and label them as mine*, *this was a real difficulty*, *but it has gradually improved over time” [Consultant Anaesthetist].*
**Clinician confidence in TIVA practice**	
Clinicians lacked confidence when using TIVA due to its complexities. Staff highlighted the potential dangers of not practicing confidently.	*“TIVA is confounded by the fact that people are less confident using it*, *and therefore*, *the anaesthetic delivery overall may not be as good as volatiles”* [Trainee Registrar]. *“This is change in how we go about our craft and the most dangerous thing in medicine is doing some that is unfamiliar to you*, *this is where you put patients at risk”* [Consultant Anaesthetist].
**Other environmental concerns**	
TIVA produces a lot more plastic waste, such as syringes, vials, and packaging. Some stated that the benefits of reducing desflurane use to the environment will be overruled by poor waste management of the increase in single-use plastic.	*“There’s not much evidence or concrete data yet on how much we are helping climate change by switching to TIVA”* [Consultant Anaesthetist]. *“The problem with TIVA is that you use a lot of plastic but also the procurement to buy in for us to be able to do things to reduce this waste effect is another issue”* [Consultant Anaesthetist]. *“To be honest*, *we’re no longer concerned about the shift to TIVA we’re more concerned about the increase in plastic waste it is creating”* [Consultant Anaesthetist].

### Theme 3: Potential barriers and enablers of ending the use of desflurane and shifting towards total intravenous anaesthesia & recommendations

The final theme presents experiences that were perceived by anaesthetists in the department affecting their transition from desflurane to TIVA. Findings fell under five sub-themes: Organisational barriers and barriers in culture; Staff members’ resistance to change; Training or staff shadowing initiatives; Organisational support and climate change; Raising awareness of climate change and connecting with national bodies to disseminate information.

#### Organisational barriers and barriers in culture

When asked about the organisational culture at this site, participants indicated that the hospital was not engaged in making changes due to logistics and lack of funding, slowing down this change to anaesthetic practice. Many clinicians stated that, when they made suggestions to the trust regarding changes, they were told there was no money.

*“Generally*, *we are not light and easy about changing things*, *there is a process. There is a lot you have to get through to make changes*, *a lot of behind the scenes work”* [Consultant Anaesthetist].

In addition, participants were sceptical whether changing individual practice would make a difference at an organisational level as they believe there is a perceived culture of indifference towards climate change at their site. Many participants stated that if a large hospital operates efficiently, making changes could disrupt these effective processes.

*“Climate indifference is the driving cultural barrier*, *we all think what we are doing won’t make the biggest change- this is the barrier”* [Consultant Anaesthetist].

#### Staff members’ resistance to change

The overarching reason why participants thought staff may not support this change came down to habit; they explained that anaesthesia is a very personal craft, and senior clinicians might find it difficult to change their practice after years of using volatiles. It is unlikely that consultants who have been practicing for years will accept the fact that they may need to be trained or retrained in a certain technique and master their practice all over again.

*“Technique is such an important*, *personal thing but there are cultural and practice factors which means that technique might be a bit slower to change”* [Trainee Registrar].

There was also evidence of a lack of understanding of the benefits of this change. Not many clinicians were familiar with the advantages of switching from desflurane to total intravenous, and many of them who knew the facts, were highly sceptical. A consultant anaesthetist stated that this idea of climate change scepticism is driven by the highly political nature of climate change. Some anaesthetic practitioners do not believe that TIVA has a lower environmental impact.

*“Discussions surrounding climate change are focused on global and national issues rather than telling us where the problems lie or what we need to focus on”* [Consultant Anaesthetist].

However, one participant stated that it was not really about the principles and benefits of the change but more about laziness, if something makes practice more difficult, clinicians are less likely to engage. It is thought that TIVA is a longer process, and this will have repercussions on waiting lists, bed flow and staff morale. Also, when different equipment and techniques need to be used, more thought must be put into what clinicians are doing.

Finally, some participants mentioned a lack of consequences which they felt led to inconsistent practices among staff. Participants provided examples of where they have observed other staff members using volatiles instead of TIVA, even in situations where they believed TIVA would be more appropriate. They explained that this is because many feel more comfortable and in control when using gas. However, this also comes down to people not wanting to be told what to do.

*“I don’t think*, *I know*, *that there are some people who will continue to use volatiles- it is their first choice”* [Consultant Anaesthetist].

#### Training or staff shadowing initiatives

A frequently mentioned enabler was training consultants who felt out of their depth while using TIVA. Participants mentioned that the focus should be to boost confidence and flexibility in practice. Suggestions were made to incorporate this into audit days to make it more accessible.

*“Anaesthesia is a very personal thing and is very dependent on the anaesthetist on what they use why and when. It is driven by the patient but also the clinical scenario and surgery. So*, *it is the person that has to change what they’re doing*, *rather than the site”* [Anaesthetist].

However, a few trainees stated that there was a difference between being taught and examined on something and being competent and confident to practise using that technique. Therefore, many suggested shadowing consultants who use TIVA. However, others suggested that TIVA has been added to the curriculum with a better focus, which must be continued and emphasised.

*“If you are not familiar you really do need someone to train you as a lack of confidence will affect efficiency and patient safety”* [Anaesthetic Trainee].

#### Organisational support and climate change

Most staff members believed that systemic barriers towards this change did not exist as this hospital is forward-thinking and supportive of change. A lot of work has been carried out by this site to reduce any potential barriers, such as making TIVA equipment readily available and, in general, the hospital was labelled as a *“pro-TIVA trust”.* Participants discussed organisational incentives to support staff to adopt changes would help increase awareness.

*“Incentivising people to think about it better*, *people don’t make change unless they have vested interest or something they care about*, *there is no incentive currently for anybody”* [Consultant Anaesthetist].

The organisational commitment to implementing change at a high level will be a significant enabler. Participants shared that as soon as the discussion of discontinuing took place, it was promptly removed from Westmoreland Street the following day. In terms of the culture of anaesthetic practitioners, the community in the UK is deemed open and innovative, according to the majority of consultants.

*“This site is the sort of place to say yes*, *this is the default answer. I wonder if this is because of funding or if someone has made a strong case and said we need x amount of pumps to make this work”* [Anaesthetist Trainee].

#### Raising awareness of climate change practice

Many were aware of the environmental impact of their work and stated that this was due to having strong climate change leadership within the department. This has made a substantial difference in supporting the shift towards TIVA. Presentations by key departmental staff members has addressed the knowledge gap in a non-discriminatory way and contributed to the shift in awareness. Individual, informal conversations between colleagues and climate change leads appeared to foster this increase in awareness.

*“Anaesthetists are very flexible*, *if they are in the know or have an interest*, *they are happy to learn and make changes and everyone willing to help one another”* [Consultant Anaesthetist].

More research would most certainly enable this shift further. Staff were interested in receiving more information relevant to this site, such as, how much their use of desflurane was contributing to GHG, so that they could put it into perspective.

*“We need a variety of things that catch interest*, *journal clubs*, *posters*, *things on the machines (stickers)*, *stop and think stickers on the volatiles- just so there is no way to get rid of it*” [Anaesthetist].

Almost all participants recommended a dashboard that displays the weekly usage of volatile gas and GHG emission. They expressed a strong desire to understand the real contributors to waste and emissions in the department, so they can identify their priorities. This could be strengthened by more signposting and visual aids around the hospital. It is crucial to advertise exactly what the changes that are being made at this site are, to make staff more conscious and engaged. For instance, having catchy “top tips” that automatically appear on staff desktops, more journal clubs, and stickers on machines to act as reminders of the negative impacts of using volatiles.

*“It would be good to know*, *what are the real contributors to waste and emissions and what do we need to prioritise”* [Consultant Anaesthetist].

Another frequently mentioned idea was the involvement of national bodies. They could play a crucial role in creating standardised information packets, participating in in governance days, and utilising social media platforms to disseminate information.

*“Ending the use of desflurane is widely accepted and people feel it is the right thing to do*, *I havent seen desflurane used anywere*, *so*, *in practice*, *people are moving away from it”* [Trainee Registrar].

## Discussion

Our findings conveyed anaesthetic practitioners’ perceptions of changing from desflurane to TIVA as part of mitigating GHG emission strategies in a London hospital anaesthesia department. Anaesthetic practitioners support the shift to TIVA due to its environmental benefits despite concerns about waste management. The invisibility of GHG emissions caused by using desflurane make it difficult for healthcare professionals to perceive associated harm. This highlights the conflation of different environmental endpoints and that clinicians’ awareness of these endpoints are still in its infancy [30]. Additionally, while TIVA avoids the direct GHG emissions associated with volatile anaesthetics like desflurane, it still has an environmental impact due to the manufacturing and disposal of consumables like plastic syringes. Therefore, using low-flow doses of sevoflurane (< 0.5 L/min fresh gas flow) can be a more environmentally friendly alternative to desflurane, given it generates much less GHG [31].

Participants conveyed drawbacks of TIVA, such as the unavailability of pumps, the increased effort involved, and perceived unreliability of brain monitoring. This aligns with previous studies, where infrequent users of TIVA discussed the added effort of performing TIVA, concerns over depth of anaesthesia and a lack of equipment as major downsides [32].

A significant advantage of using TIVA in our sample was reduced postoperative nausea, which is a well-established finding across the literature [33]. However, other studies have looked further into the wider benefits of this advantage, which found that less postoperative nausea necessitates fewer antiemetics, thereby representing a saving in drug costs, and preventing patient dissatisfaction [34].

In addition, our findings suggested that staff recognised a significant organisational benefit of using desflurane to facilitate faster turnaround of patients due to quicker wake-up and fewer complications. However, although staff perceived these benefits, other studies have found that discharge times were not necessarily improved [17].

In terms of potential barriers to this transition, participants anticipated resistance from some staff members who would not support the idea of changing their practice due to habit, laziness, and a lack of understanding of the benefits. There was strong emphasis on the likelihood that senior clinicians, who have spent years perfecting their skills using volatile agents, are likely to resist this change. This finding is supported by a study [32] which found younger clinicians are more amenable to respecting organisational preferences as older clinicians often define these preferences and are less likely to abandon habits.

Several participants recommended the need for more information on the damage their practice is causing, including a dashboard that informs of the volatile gas that has been used each week and the level of GHG emission. This highlights the idea of visibility. Staff acknowledge that making the consequences of their practice more visible will help them engage with climate change mitigation. This is supported by the Royal College of Anaesthetists [35] who demonstrated that if sustainability leads within anaesthesia provide such data, it not only raises awareness, but also alters departmental behaviours.

Anaesthetic practitioners in this department recognised and took steps to address their climate change practices. This supports the need for greater deployment of incentives, incorporating the social influences such as shadowing that anaesthetic practitioners see as potentially effective. Our findings also indicate a need for training of anaesthetic practitioners who felt less confident using TIVA. Training programmes have been proven successful in changing the beliefs and behaviours of healthcare workers [8]. Finally, it is important to recognise this is a complex issue, the broader concerns related to volatile anaesthetics reflect the interconnected nature of volatile practices and their environmental impact.

### Strengths and limitations

This study contributes to the limited qualitative literature on anaesthetics’ perspectives of reducing carbon emissions in healthcare settings. This study gave voices to the individuals directly involved in the transition from desflurane to TIVA, providing valuable insights for hospital decision-makers. Qualitative research allows evaluators to understand the organisational and cultural context in which behaviours occur and enable further research and provide insights for other similar departments. This study can help embed learning from evaluations aimed at promoting sustainable healthcare interventions into practice.

The findings of this study must be interpreted in relation to its limitations. Firstly, while the initial recruitment strategy aimed to be inclusive of different professional roles, our sample consisted of mostly consultant anaesthetists, potentially biasing results due to self-selection bias whereby those who had an interest in climate change were likely to have agreed to participate. In addition, the findings of the study may not be directly transferable to other hospitals or healthcare settings due to the unique specificities of each hospital. The limited sample size can further limit the transferability of the findings, emphasising the need for future research with a more diverse sample such as junior trainees, climate sceptics and within different hospital contexts. This study used qualitative research, many of our themes warrant more in-depth exploration. A rapid qualitative appraisal can have limitations in comprehensively addressing multiple aspects of a complex topic. For example, this study did not specifically explore the potential oncological benefits of propofol over volatile anaesthetics, which could be considered in future research or clinical practice decisions. In addition, the availability or utilisation of monitoring equipment such as BIS and SedLine monitors were not assessed, which could have provided additional insights into clinical practices. Finally, the study focused on transitioning from desflurane to TIVA, overlooking all aspects of halogenated gases, pollution and other potential strategies for reducing gas usage or monitoring the depth of anaesthesia.

## Research and clinical implications

To promote the sustainable use of inhalational anaesthesia, the hospital built a Best Practice Advisory into the Electronic Health Record System. This system triggers a reminder if the anaesthetist is using fresh gas flows of > 1 L per minute. While this intervention addresses fresh gas flow rates, it does not directly address our primary aim, which was to replace desflurane with TIVA. However, it represents an additional day-to-day strategy to further reduce GHG emissions from inhalation anaesthesia. Future research is needed for the evaluation of such interventions that target the carbon footprint of healthcare, including the replacement of desflurane with TIVA.

Approaches to understanding clinical behaviour towards reducing or shifting carbon-intensive actions are rarely reported but have clear potential benefits, such as overcoming obstacles due to organisational barriers and fostering a culture of sustainable anaesthesia practice. The decision to stop using desflurane in this hospital site was transformative because it developed a shared purpose. The collective engagement was a cultural catalyst to drive the department together toward the delivery of more sustainable anaesthesia in the future,

This study explored specific sub-interventions, future research should explore the perceptions, barriers, and enablers of each sub-intervention separately, whilst also investigating variations in engagement across other Trusts and around the country. It is important to avoid dichotomies between environmental sustainability and patient safety, there is a need for a more nuanced approach that separately examines each intervention. Approaches to waste management within anaesthesia departments should also be explored, as participants believed this would lead to a larger culture shift.

The diversity of recommendations for transitioning to TIVA and suggested strategies indicate that a variety of approaches are potentially helpful. These include training programmes for clinicians to enhance their understanding of sustainable practices and the environmental impact of anaesthetic practices, while also addressing misconceptions and providing evidence-based practice.

### Electronic supplementary material

Below is the link to the electronic supplementary material.

Supplementary Material 1

## Data Availability

The datasets generated during and/or analysed during the current study are available from the corresponding author on reasonable request.

## Abbreviations

HCWs: Healthcare workers
HRA: Health Research Authority
NHS: National Health Service
RaDAR: Rapid and Rigorous Qualitative Data Analysis
RAP: Rapid Appraisal Procedures
RREAL: Rapid Research and Evaluation Lab
TIVA: Total Intravenous Anaesthesia
WHO: World Health Organisation

## References

[1] The World Health Organisation. 2021. Climate change and health.

[2] Bernat M, Boyer A, Roche M, Richard C, Bouvet L, Remacle A, et al. Reducing the carbon footprint of general anaesthesia: a comparison of total intravenous anaesthesia vs. a mixed anaesthetic strategy in 47,157 adult patients. Anaesthesia. 2024;79(3):309–17. 38205529 10.1111/anae.16221

[3] NHS England. 2019. The NHS long-term plan. London EA. https://www.longtermplan.nhs.uk

[4] Health and Care Act. 2022, c. 31. https://www.legislation.gov.uk/ukpga/2022/31/contents/enacted

[5] NHS, Greener. NHS campaign to tackle climate ‘health emergency’. 2020.

[6] Batcup C, Breth-Petersen M, Dakin T, Barratt A, McGain F, Newell BR, et al. Behavioural change interventions encouraging clinicians to reduce carbon emissions in clinical activity: a systematic review. BMC Health Serv Res. 2023;23(1):384. 37081553 10.1186/s12913-023-09370-2 PMC10116654

[7] Sulbaek Andersen M, Sander S, Nielsen O, Wagner D, Sanford T Jr, Wallington T. Inhalation anaesthetics and climate change. Br J Anaesth. 2010;105(6):760–6. 20935004 10.1093/bja/aeq259

[8] Charlesworth M, Swinton F. Anaesthetic gases, climate change, and sustainable practice. Lancet Planet Health. 2017;1(6):e216–7. 29851604 10.1016/S2542-5196(17)30040-2

[9] Vollmer MK, Reimann S, Hill M, Wyss SA, Henne S, Emmenegger L. Halogenated greenhouse gases at Jungfraujoch.

[10] MacNeill AJ, Lillywhite R, Brown CJ. The impact of surgery on global climate: a carbon footprinting study of operating theatres in three health systems. Lancet Planet Health. 2017;1(9):e381–8. 29851650 10.1016/S2542-5196(17)30162-6

[11] Thiel CL, Eckelman M, Guido R, Huddleston M, Landis AE, Sherman J, et al. Environmental impacts of surgical procedures: life cycle assessment of hysterectomy in the United States. Environ Sci Technol. 2015;49(3):1779–86. 25517602 10.1021/es504719g PMC4319686

[12] Fawzy S, Osman AI, Doran J, Rooney DW. Strategies for mitigation of climate change: a review. Environ Chem Lett. 2020;18(6):2069–94. 10.1007/s10311-020-01059-w

[13] Roa L, Velin L, Tudravu J, McClain CD, Bernstein A, Meara JG. Climate change: challenges and opportunities to scale up surgical, obstetric, and anaesthesia care globally. Lancet Planet Health. 2020;4(11):e538–43. 33159881 10.1016/S2542-5196(20)30247-3

[14] Al-Rifai Z, Mulvey D. Principles of total intravenous anaesthesia: practical aspects of using total intravenous anaesthesia. BJA Educ. 2016;16(8):276–80. 10.1093/bjaed/mkv074

[15] Masud MM, Al-Amin AQ, Junsheng H, Ahmed F, Yahaya SR, Akhtar R, et al. Climate change issue and theory of planned behaviour: relationship by empirical evidence. J Clean Prod. 2016;113:613–23. 10.1016/j.jclepro.2015.11.080

[16] Hathaway J, Maibach EW. Health implications of climate change: a review of the literature about the perception of the public and health professionals. Curr Environ Health Rep. 2018;5:197–204. 29423661 10.1007/s40572-018-0190-3 PMC5876339

[17] White S, Shelton C, Gelb A, Lawson C, McGain F, Muret J, et al. Principles of environmentally-sustainable anaesthesia: a global consensus statement from the World Federation of Societies of Anaesthesiologists. Anaesthesia. 2022;77(2):201–12. 34724710 10.1111/anae.15598 PMC9298028

[18] Quitmann C, Sauerborn R, Danquah I, Herrmann A. Climate change mitigation is a hot topic, but not when it comes to hospitals’: a qualitative study on hospital stakeholders’ perception and sense of responsibility for greenhouse gas emissions. J Med Ethics. 2023;49(3):204–10. 35459742 10.1136/medethics-2021-107971 PMC9985738

[19] Cliff BQ, Avanceña ALV, Hirth RA, Lee SD. The impact of choosing wisely interventions on Low-Value Medical Services: a systematic review. Milbank Q. 2021;99(4):1024–58. 34402553 10.1111/1468-0009.12531 PMC8718584

[20] Johnson GA, Vindrola-Padros C. Rapid qualitative research methods during complex health emergencies: a systematic review of the literature. Soc Sci Med. 2017;189:63–75. 28787628 10.1016/j.socscimed.2017.07.029

[21] Vindrola-Padros C. Doing Rapid qualitative research. SAGE; 2020.

[22] Nevedal AL, Reardon CM, Opra Widerquist MA, Jackson GL, Cutrona SL, White BS, et al. Rapid versus traditional qualitative analysis using the Consolidated Framework for Implementation Research (CFIR). Implement Sci. 2021;16(1):1–12. 34215286 10.1186/s13012-021-01111-5 PMC8252308

[23] Sobo EJ, Simmes DR, Landsverk JA, Kurtin PS. Rapid assessment with qualitative telephone interviews: lessons from an evaluation of California’s healthy families program & Medi-Cal for children. Am J Evaluation. 2003;24(3):399–408.

[24] Beebe J. Rapid assessment process: an introduction. Rowman Altamira; 2001.

[25] Leech BL. Interview methods in political science. PS-WASHINGTON-. 2002;35(4):663–4.

[26] Vindrola-Padros C, Johnson GA. Rapid Techniques in Qualitative Research: a critical review of the literature. Qual Health Res. 2020;30(10):1596–604. 32667277 10.1177/1049732320921835

[27] Nevedal AL, Reardon CM, Opra Widerquist MA, Jackson GL, Cutrona SL, White BS, et al. Rapid versus traditional qualitative analysis using the Consolidated Framework for Implementation Research (CFIR). Implement Sci. 2021;16(1):67. 34215286 10.1186/s13012-021-01111-5 PMC8252308

[28] Gale NK, Heath G, Cameron E, Rashid S, Redwood S. Using the framework method for the analysis of qualitative data in multi-disciplinary health research. BMC Med Res Methodol. 2013;13(1):117. 24047204 10.1186/1471-2288-13-117 PMC3848812

[29] Watkins DC. Rapid and Rigorous qualitative data analysis:the RADaR technique for Applied Research. Int J Qualitative Methods. 2017;16(1):1609406917712131. 10.1177/1609406917712131

[30] Varughese S, Ahmed R. Environmental and occupational considerations of anesthesia: a narrative review and update. Anesth Analg. 2021;133(4):826. 33857027 10.1213/ANE.0000000000005504 PMC8415729

[31] Kostrubiak M, Vatovec CM, Dupigny-Giroux LA, et al. Water Pollution and environmental concerns in Anesthesiology. J Med Syst. 2020;44:169. 10.1007/s10916-020-01634-2 32794038 10.1007/s10916-020-01634-2

[32] Wong G, Choi S, Tran D, Kulkarni H, Irwin M. An international survey evaluating factors influencing the use of total intravenous anaesthesia. Anaesth Intensive Care. 2018;46(3):332–8. 29716493 10.1177/0310057X1804600312

[33] Elbakry AE, Sultan WE, Ibrahim E. A comparison between inhalational (desflurane) and total intravenous anaesthesia (propofol and dexmedetomidine) in improving postoperative recovery for morbidly obese patients undergoing laparoscopic sleeve gastrectomy: a double-blinded randomised controlled trial. J Clin Anesth. 2018;45:6–11. 10.1016/j.jclinane.2017.12.001 29223575 10.1016/j.jclinane.2017.12.001

[34] Sneyd JR, Holmes KA. Inhalational or total intravenous anaesthesia: is total intravenous anaesthesia useful and are there economic benefits? Curr Opin Anesthesiology. 2011;24(2):182–7. 10.1097/ACO.0b013e328343f3ac 21252648

[35] Pierce J. M. The environment, the gas bill and the route to sustainable anaesthesia. Royal College of Anaesthetists; 2013.

