# Supplementary material for: Anaesthesia and climate change: time to wake up? A rapid qualitative appraisal exploring the views of anaesthetic practitioners regarding the transition to TIVA and the reduction of desflurane
Source: BMC Anesthesiol. 2024 Aug 30;24:300. doi: 10.1186/s12871-024-02693-5 (PMC11363533; doi:10.1186/s12871-024-02693-5)
Supplement: Supplementary file 1 — Supplementary Material 1 [file 12871_2024_2693_MOESM1_ESM.docx]

INTERVIEW TOPIC GUIDE

1. Background: Can you tell me about your role? (daily tasks, responsibilities etc)
2. Background: Can you tell me about the use of vaporisers and intravenous anaesthesia? (When are they used, why? Are they often used together?)
3. What are your views in relation to **climate change**?
4. How is **climate change perceived** by your colleagues?
   1. Is it considered a critical issue?
   2. Is it something that is discussed at all?
   3. In what ways do you think the hospital contributes to climate change? (In terms of waste, pollution, carbon footprint).
   4. Are you aware that a large proportion of the hospital’s carbon footprint is from anaesthetic gases?
   5. Are there any specific climate change mitigation strategies in anaesthesia that would be effective, in your opinion?
5. What do you think about the **change** in anaesthesia with respect to ending desflurane use and increasing TIVA anaesthesia?
6. How is the **change** in anaesthesia with respect to ending desflurane use and increasing TIVA anaesthesia **perceived by** your colleagues?
7. What kind of impact do you think this change will have on **patient** care?
   1. What are the advantages and disadvantages to patient care? (Will this change create any pre-operative/ post-operative challenges)?
   2. Recovery (haemodynamic stability, predictable and fast recovery, post-operative nausea, and vomiting), side-effects, efficacy of the anaesthesia, safety, risks?
   3. Are there any circumstances under which desflurane would have to be used? (aka. Cases where it cannot be replaced?)
   4. Is desflurane needed to safely deliver care (to anaesthetise) patients?
8. What type of impact will this change have on your practice?
   1. How will it impact **care delivery**? (How would administering the anaesthetic change? Different staff needed?)
   2. Is TIVA more **technical** to administer? (Is more skill required; does it take longer to administer)?
   3. Will this change create different dynamics in the operating theatres?
   4. Would changes need to be made regarding **training** new anaesthetists/ ODPS, theatre staff?
   5. How else can you maintain anaesthesia if vaporises are not used?
   6. Will there be patients that object not having inhalation gas?
   7. Do you have any concerns?
      1. If yes: Do you have the ability to voice these concerns?
9. Is there anything else you would like to mention about plans to change anaesthetic practice that we haven’t yet asked you about today?

Thank you very much for your time.
